# Clinical impact of splicing in neurodevelopmental disorders

**DOI:** 10.1186/s13073-020-00737-2

**Published:** 2020-04-24

**Authors:** Stephan J. Sanders, Grace B. Schwartz, Kyle Kai-How Farh

**Affiliations:** 1 Department of Psychiatry and UCSF Weill Institute for Neurosciences, University of California, San Francisco, San Francisco, CA 94158 USA; 2 Illumina Artificial Intelligence Laboratory, Illumina, Inc., San Diego, CA USA

**Keywords:** Gene splicing, Isoform, SpliceAI, Autism spectrum disorder, Developmental delay, Clinical exome sequencing, Cryptic splice site, Canonical splice site, Polypyrimidine tract, Antisense oligonucleotide

## Abstract

Clinical exome sequencing is frequently used to identify gene-disrupting variants in individuals with neurodevelopmental disorders. While splice-disrupting variants are known to contribute to these disorders, clinical interpretation of cryptic splice variants outside of the canonical splice site has been challenging. Here, we discuss papers that improve such detection.

## Splicing disruption in human disorders

Gene-disrupting genetic variants frequently lead to neurodevelopmental disorders, including developmental delay and autism spectrum disorder (ASD), when they occur in one of the several hundred genes associated with these disorders [1, 2]. Many of these variants are de novo, observed in the affected child, but not in either parent, and capable of mediating substantial risk for neurodevelopmental disorders. Such variants alter the quantity or quality of the encoded proteins, through deletions, premature stop codons, or missense variants. In this commentary, we consider the impact of an additional class of gene-disrupting variants that act by altering gene splicing. Three papers outline improvements in detecting splice-disrupting variants [3–5], and applying these methods predicts cryptic splicing variants in genes associated with neurodevelopmental disorders in about 0.5% of cases and no controls [1, 2].

## Splicing motifs and mechanisms

**Fig. 1:**
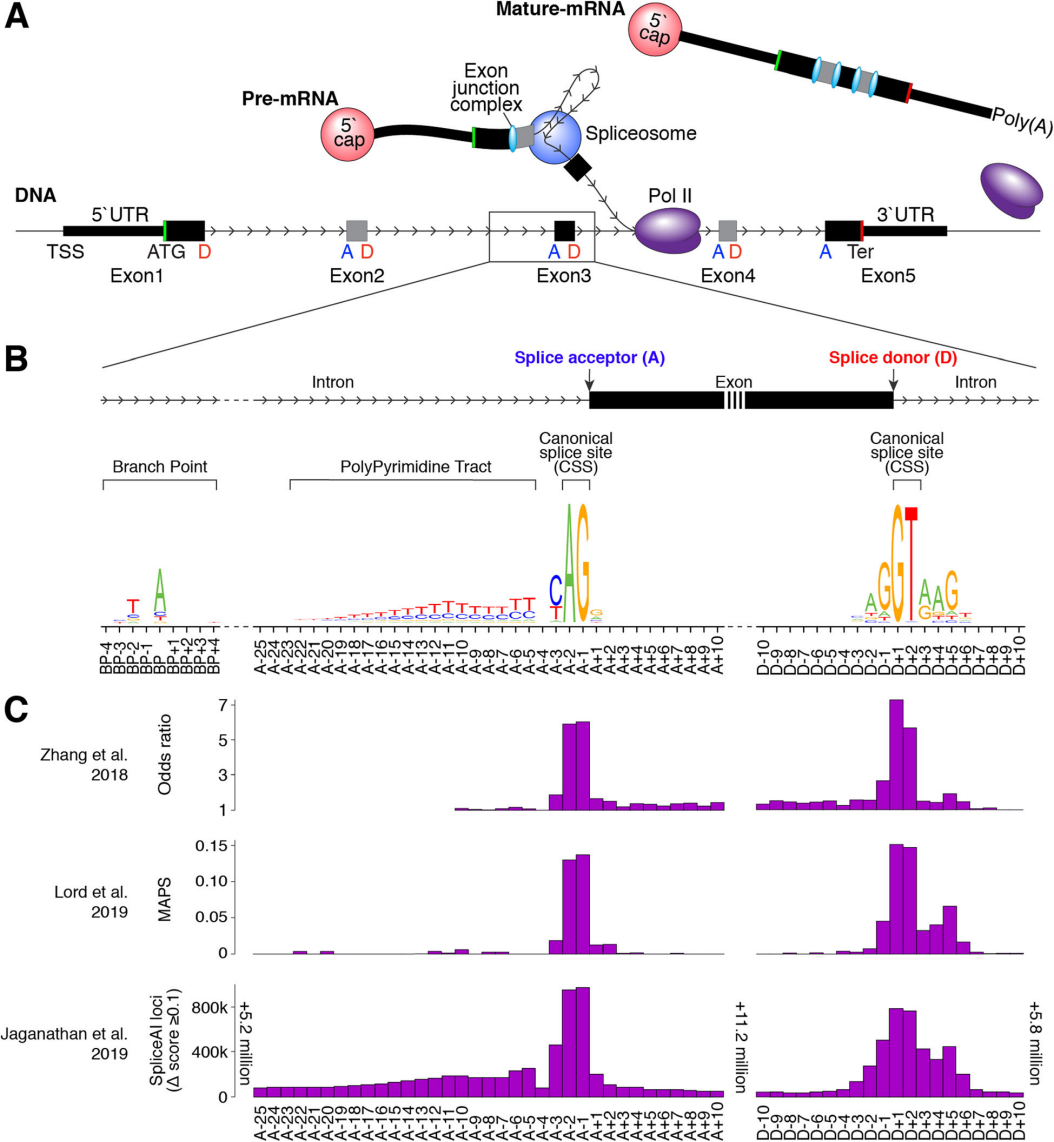
Overview of the splicing region. **a** The spliceosome attaches to pre-mRNA as it is transcribed from DNA, removing introns and leaving an exon junction complex upstream. The mature mRNA can migrate out of the nucleus for translation. **b** Motifs of the polypyrimidine tract, acceptor, and donor, calculated from all protein-coding exons. **c** Odds ratios of observed and expected variant frequencies around the splice site based on ExAC exome sequencing data in Zhang et al. [4]. Lord et al. [3] use the same ExAC data to calculate the mutability-adjusted proportion of singletons (MAPS) across splicing regions, which is higher at nucleotides intolerant of variation. Jaganathan et al. [5] developed SpliceAI, a neural network for predicting the impact of variants on splicing across the genome; the number of potential variants with a Δ score ≥ 0.1 is shown across splicing regions. Abbreviations: TSS, transcription start site; UTR, untranslated region; A, acceptor; D, donor; Pol II, polymerase II; Ter, termination codon; Poly(A), polyadenylation

Splicing is a key process in eukaryotic cells. After transcription, a nascent pre-mRNA must be converted into a mature mRNA that can serve as a template for protein translation. This involves the removal of introns from the pre-mRNA, usually by the major spliceosome, through splicing (Fig. 1a). Critical to this process are the two-nucleotide “essential” or “canonical” splice sites (CSS) at either side of exons: an “AG” motif upstream of the acceptor site (A, also called the 3′ splice site), at positions A-1 and A-2, and a “GT” motif downstream of the donor site (D, also called the 5′ splice site), at positions D+1 and D+2 (Fig. 1b).

Along with the CSS, other DNA features are known to determine splicing behavior, including several motifs representing binding targets of the small nuclear ribonucleoproteins (snRNPs) that make up the major spliceosome. Motif analysis across exons (Fig. 1b) has identified broader “CAG” and “AGGTAAGT” motifs at the acceptor and donor, respectively, as well as the polypyrimidine tract, characterized by enrichment of thymine and cytosine upstream of the acceptor (A-5 to A-40). Upstream of the polypyrimidine tract is the branch point (A-10 to A-50, median A-25) with a “TNA” motif (Fig. 1b). In the major spliceosome, U1 snRNPs bind to the donor site, U2 snRNPs bind to the branch point, and the U2AF protein binds to the polypyrimidine tract and acceptor site [6].

## Variation at canonical splice sites

Noncoding genetic variants that disrupt the CSS of critical genes are a known cause of human genetic diseases, including neurodevelopmental disorders [1, 2]. Improper splicing can lead to exon skipping or novel splice sites, both of which can alter the reading frame of protein-coding genes. Alternatively, intron retention incorporates noncoding DNA, which often contains stop codons, into the mature RNA. Consequently, identifying CSS variants in genes with known disease associations is a routine practice in clinical exome sequencing, in which they are treated as protein-truncating variants (PTVs) along with premature stop codons or frameshift variants [7].

Out of 1863 de novo variants identified in individuals with neurodevelopmental disorders [2], the 296 CSS variants account for 16% of PTVs, 637 premature stop codons account for 34%, and 930 frameshift insertions or deletions account for 50%. To quantify the contribution of these de novo variants to disorders, we consider the frequency of variants in 3230 protein-coding genes that are predicted to be “PTV-intolerant,” based on fewer than expected PTVs in whole-exome sequencing data from over 60,000 individuals (expressed statistically as a probability loss-of-function intolerant (pLI) score ≥ 0.9) [8]. Comparing differences in the rate of de novo PTVs in these PTV-intolerant genes between cases and controls [1, 2], we estimate that de novo PTVs contribute to 5% of ASD cases and 16% of developmental delay cases. Since 16% of PTVs are in the CSS, this equates to 0.8% of ASD cases and 2.6% of developmental delay cases due to splicing disruption at the CSS.

## Variation at cryptic splice sites

Exonic or intronic splice-disrupting variants outside of the CSS are commonly referred to as cryptic splice variants, due to the challenge of identifying them. The below articles focus on improving clinical interpretation of these cryptic splice sites in neurodevelopmental disorders by leveraging exome sequencing data from population samples [3, 4] or deep learning methods [5]. Zhang et al. [4] use exome sequencing data from over 60,000 population samples from the Exome Aggregation Consortium (ExAC) [8] to assess the observed versus expected number of variants in the 10 nucleotides flanking the acceptor and donor sites in PTV-intolerant genes. They highlight six non-CSS nucleotides that are intolerant of variation (Fig. 1c) and validate splicing dysregulation in four of these (D-1, D+4, D+5, D+6) using paired whole-genome and RNA sequencing from GTEx [9]. Such cryptic splice de novo variants were observed in 0.2% of ASD cases and 0.2% of developmental delay cases. Lord et al. also use ExAC data [3] to highlight two nucleotides that are intolerant of variation (D-1, D+5, Fig. 1c). The D+5 site is also enriched for de novo variants in cases of developmental delay in genes associated with this disorder, as was the polypyrimidine tract when all nucleotides (A-5 to A-25) were considered together. By integrating phenotype data, they identified 18 likely diagnostic de novo variants in 7833 cases (0.2%). Functional assessment of splicing using a minigene assay validated six of the seven likely diagnostic variants that were tested (86%).

Jaganathan et al., which includes the authors of this commentary [5], describe the SpliceAI algorithm, a neural network that predicts the impact of cryptic splice variants based on a pre-mRNA sequence. The network, trained on 10,000 nucleotides of human genomic sequence around 260,000 known splice sites from GENCODE, is used to calculate the SpliceAI Δ score by considering the difference in predicted splicing between reference and variant sequence. Scores range from 0 to 1 with high scores more likely to alter splicing (Fig. 1c). Assessing performance in the paired whole-genome and RNA sequencing data from GTEx [9] identifies splicing disruption proportional to the Δ score (i.e., 20% at 0.2; 80% at 0.8) with higher sensitivity and specificity than prior algorithms [5]. High depth RNA-seq of ASD patient-derived lymphoblastoid cell lines validated 21 of 28 (75%) de novo variants predicted to alter splicing (Δ score, 0.10–0.99; median 0.58), including variants in the ASD-associated genes *TCF4* and *KDM6B* [2]. Of note, analysis of GTEx also revealed widespread tissue-specific splicing, which may lead such validation to underestimate the true accuracy. An excess of de novo variants predicted to alter splicing (Δ score ≥ 0.1) was observed in both developmental delay and ASD, compared to controls. Considering only genes previously associated with neurodevelopmental disorders, de novo variants at cryptic splice sites were observed in 23 out of 3953 ASD cases (0.6%), 21 out of 4293 developmental delay cases (0.5%), and none of the 2073 controls [1, 2].

Overall, SpliceAI predicts about 7-fold more cryptic splice site variants than the other two approaches because it is not limited to specific nucleotides (e.g. D+5), includes splice sites further from the exons, and evaluates each splice site individually. Considering variants assessed consistently between these three methods, SpliceAI predicts all four “likely diagnostic” variants in Lord et al. and 10 of the 18 variants (56%) highlighted by Zhang et al.

With these improvements in detection [3–5], we propose that de novo variants at cryptic splice sites identified in exome or genome sequencing of individuals with neurodevelopmental disorders should undergo clinical evaluation in a manner similar to deleterious missense variants. Such evaluation would incorporate evidence from gene association studies, pLI scores, and consistency of phenotype [7].

## Prevalence of splicing disruption in neurodevelopmental disorders and therapeutic potential

Using the SpliceAI estimates, splicing disruption by de novo variants in PTV-intolerant genes underlies at least 1.4% of ASD cases (0.8% CSS and 0.6% cryptic, see estimates above) and 3.1% of developmental delay cases (2.6% CSS and 0.5% cryptic, see estimates above). These estimates are equivalent to about 20,000 ASD cases 18 years-of-age or below in the USA and 21,000 equivalent developmental delay cases. Inclusion of more genes (PTV-tolerant, noncoding), whole-genome sequencing to identify deep intronic variants missed by exome sequencing, and consideration of homozygous and heterozygous inherited variation will only increase these estimates.

While splicing variants contribute to thousands of cases of neurodevelopmental disorders, they may offer opportunities for novel therapeutic targets. The success of the FDA-approved antisense oligonucleotide (ASO) Nusinersen to modify splicing behavior, resulting in life-saving clinical improvement in patients with spinal muscular atrophy [10], sets a precedent for treating central nervous system disorders via splicing mechanisms. Such a therapy would need to be developed specifically for each splicing variant in most neurodevelopmental disorders [11]. Key research milestones will include assessing the fraction of splicing variation that can be rescued by ASOs, efficient methods to design and test ASOs, and assessment of the extent of rescue in vivo. These approaches may provide the first insights into whether gene therapy can modify the symptoms of ASD and developmental delay, potentially providing a route to treatment for thousands of individuals with splicing variants and de-risking more complicated approaches to gene therapy that could be applicable in larger populations.

## Data Availability

All data are provided by the manuscripts cited in this commentary. SpliceAI Δ scores for SNVs are available at https://basespace.illumina.com/s/5u6ThOblecrh, and the SpliceAI code is available at https://github.com/Illumina/SpliceAI.

## Abbreviations

A: Acceptor
AI: Artificial intelligence
ASD: Autism spectrum disorder
ASO: Antisense oligonucleotide
CSS: Canonical splice site
D: Donor
DNA: Deoxyribonucleic acid
ExAC: Exome Aggregation Consortium
FDA: Food and Drug Administration
GTEx: Genotype-Tissue Expression Project
KDM6B: Lysine demethylase 6B
RNA: Ribonucleic acid
pLI score: Probability loss-of-function intolerant score
Pol II: Polymerase II
Poly(A): Polyadenylation
PTV: Protein-truncating variant
snRNP: Small nuclear ribonucleoproteins
TCF4: Transcription factor 4
Ter: Termination codon
TSS: Transcription start site
UTR: Untranslated region
